# Current NAFLD guidelines for risk stratification in diabetic patients have poor diagnostic discrimination

**DOI:** 10.1038/s41598-020-75227-x

**Published:** 2020-10-27

**Authors:** Valentin Blank, David Petroff, Sebastian Beer, Albrecht Böhlig, Maria Heni, Thomas Berg, Yvonne Bausback, Arne Dietrich, Anke Tönjes, Marcus Hollenbach, Matthias Blüher, Volker Keim, Johannes Wiegand, Thomas Karlas

**Affiliations:** 1 Division of Gastroenterology, Department of Medicine II, Leipzig University Medical Center, Liebigstraße 20, 04103 Leipzig, Germany; 2 Integrated Research and Treatment Center (IFB) AdiposityDiseases, University of Leipzig, Philipp-Rosenthal-Str. 27, 04103 Leipzig, Germany; 3 Clinical Trial Centre Leipzig, University of Leipzig, Härtelstraße 16/18, 04107 Leipzig, Germany; 4 Division of Hepatology, Department of Medicine II, Leipzig University Medical Center, Liebigstraße 20, 04103 Leipzig, Germany; 5 Division of Angiology, Leipzig University Medical Center, Liebigstraße 20, 04103 Leipzig, Germany; 6 Division of Visceral, Transplantation, Thorax and Vascular Surgery, Section of Bariatric Surgery, Leipzig University Medical Center, Liebigstraße 20, 04103 Leipzig, Germany; 7 Division of Endocrinology and Nephrology, Leipzig University Medical Center, Liebigstraße 20, 04103 Leipzig, Germany

**Keywords:** Diabetes complications, Non-alcoholic fatty liver disease, Non-alcoholic steatohepatitis, Risk factors

## Abstract

Patients with type 2 diabetes (T2D) are at risk for non-alcoholic fatty liver disease (NAFLD) and associated complications. This study evaluated the performance of international (EASL-EASD-EASO) and national (DGVS) guidelines for NAFLD risk stratification. Patients with T2D prospectively underwent ultrasound, liver stiffness measurement (LSM) and serum-based fibrosis markers. Guideline-based risk classification and referral rates for different screening approaches were compared and the diagnostic properties of simplified algorithms, genetic markers and a new NASH surrogate (FAST score) were evaluated. NAFLD risk was present in 184 of 204 screened patients (age 64.2 ± 10.7 years; BMI 32.6 ± 7.6 kg/m^2^). EASL-EASD-EASO recommended specialist referral for 60–77% depending on the fibrosis score used, only 6% were classified as low risk. The DGVS algorithm required LSM for 76%; 25% were referred for specialised care. The sensitivities of the diagnostic pathways were 47–96%. A simplified referral strategy revealed a sensitivity/specificity of 46/88% for fibrosis risk. Application of the FAST score reduced the referral rate to 35%. This study (a) underlines the high prevalence of fibrosis risk in T2D, (b) demonstrates very high referral rates for in-depth hepatological work-up, and (c) indicates that simpler referral algorithms may produce comparably good results and could facilitate NAFLD screening.

## Introduction

Non-alcoholic fatty liver disease (NAFLD) is a growing epidemic, which is associated with obesity and the metabolic syndrome^1,2^, now affecting 25% of the world’s population^3^. Especially NAFLD patients with obesity and type 2 diabetes are at high risk for inflammatory disease progression (non-alcoholic steatohepatitis, NASH)^4^. In such patients, the stage of liver fibrosis is the strongest predictor for overall survival and liver-related morbidity and mortality^5–7^. This disease burden is expected to increase in the future and pose serious challenges to health-care systems^8^.

Unfortunately, to date, the risk stratification of NAFLD patients relies on sophisticated histological assessment, which itself has poor inter-rater reliability and is contra-indicated in patients suspected for liver injuries^4,9–11^. However, to address the growing prevalence of fatty-liver disease adequately, easy to perform, economically viable and widely available non-invasive methods for disease staging and grading are required^12^. Over the last decade, ultrasound-based methods, namely liver elastography, serum-based fibrosis markers and anthropometry-based scores have been proposed for risk stratification of NAFLD^13^. Elastography exploits the fact that the liver hardens with increasing fibrosis, a property that can be measured by assessing the liver’s Young’s modulus. The available methods have their advantages and disadvantages and none of them has sufficient diagnostic properties to warrant universal acceptance^13,14^. Hence research has focused on combinations of methods to improve diagnostic performance that often include laboratory parameters, conventional sonography and liver stiffness measurements (LSM) with elastography^15^. The evaluation of such step-wise algorithms is often limited by the high prevalence of advanced disease inherent to biopsy-controlled studies, rendering extrapolation to the screening setting bold at best^16,17^. Nonetheless, national and international guidelines recommend complex diagnostic workups, especially for screening purposes in primary care including the important field of diabetology^4,18^. Hitherto, these guidelines have not been widely adopted^19,20^ and concerns regarding feasibility in clinical practice have been voiced^12,21^.

Therefore, we designed a study to evaluate the current screening recommendations in the EASL-EASD-EASO and German guidelines (DGVS)^4,18^ for identifying patients at risk for NAFLD, especially regarding referral rates. We further identified factors that could facilitate referral decisions in primary care including a novel NASH surrogate score^22^.

## Patients and methods

### Ethical statement

The study was performed in accordance with the guidelines for good clinical practice (E6/R1) and the ethical guidelines of the Helsinki Declaration. The study was approved by the local ethical committee (University of Leipzig, registration number 035/17-ek) and registered in the German Clinical Trials Register (DRKS00012281). Informed written consent was obtained from all participants.

### Design overview and patients

This was a prospective cross-sectional study designed to evaluate the performance of current guideline recommendations on NAFLD risk assessment in patients with diabetes. The corresponding analysis comprised referral rates for each guideline risk class along with the association according to fibrosis risk. In addition, simplified algorithms were identified, and their diagnostic properties evaluated. The potential use of genetic risk markers and a new NASH surrogate (FAST score^22^) were analysed.

From April 2017 to May 2018, consecutive adult patients (≥ 18 years) with established diagnosis of type 2 diabetes were invited to participate in the study. We considered patients at our hospital within a treatment programme for type 2 diabetes and its sequelae or those referred from specialized collaborating type 2 diabetes out-patient centres. Patients were eligible for inclusion when diagnosis of type 2 diabetes was established according to national guideline recommendations^23^. In brief, HbA1c ≥ 6.5% or fasting plasma glucose concentration ≥ 7.0 mmol/l at the time of initial diagnosis were required for type 2 diabetes diagnosis^23,24^. Exclusion criteria comprised liver transplantation or major liver surgery, pregnancy or lactation, malignant diseases with restricted life expectancy, or other types of diabetes. Patients who reported increased alcohol consumption (women > 140 g/week, men > 210 g/week, respectively^4,25,26^) were excluded from the final analysis.

### Study examination

All patients underwent a thorough interview including medical history of type 2 diabetes and liver diseases as well as alcohol, smoking, and caffeine consumption habits. On the same day, a clinical examination including assessment weight and height, abdominal ultrasound examination, liver stiffness measurement (LSM) with vibration controlled transient elastography (VCTE) combined with measurement of controlled attenuation parameter (CAP) and laboratory assessment were performed. All patients fasted overnight prior to the study examinations.

### Ultrasound examination

A standardized abdominal ultrasound examination was performed by a certified experienced examiner (VB). A high-end ultrasound device (Toshiba Aplio 500, software version AB_V7.00*R003, Canon Medical Systems, Tustin, USA) equipped with a linear and curved array probe was used. Presence of hepatic steatosis was defined by a bright echo pattern of the liver parenchyma compared to the right kidney^4^. Biliary obstruction, liver congestion due to right heart failure, presence of ascites, or focal liver lesions at the LSM measurement site were ruled out in all patients.

### Vibration controlled transient elastography (VCTE)

All participants underwent LSM with VCTE (FibroScan; Echosens, Paris, France), using the appropriate probe (M or XL-probe, 3.5/2.5 MHz, respectively), defined by the skin-to-liver-capsule distance (M-probe ≤ 25 mm; XL-probe > 25 mm). VCTE was performed by a trained certified examiner in accordance with guidelines^14^ as described previously^27^. Elevated LSM indicated suspicion of significant liver fibrosis. An intermediate fibrosis risk was defined by LSM between 7.9 and 9.6 kPa for the M-probe (7.2–9.3 kPa for the XL-probe^18^). In addition to the LSM value, the VCTE device calculates CAP as a surrogate for severity of hepatic steatosis, expressed in dB/m^28^. We used accepted cut-offs to define steatosis grading^29^.

### Laboratory analysis and laboratory-based fibrosis scores

Same day blood samples were taken, unless data were available from within eight weeks. The following parameters were recorded: standard blood count, alanine and aspartate aminotransferases (ALT and AST), gamma-glutamyl-transferase (GGT), glycohemoglobin (HbA1c), high-density lipoprotein (HDL) and triglycerides. In all patients, fasting glucose and insulin were determined after overnight fasting on the day of study inclusion.

Laboratory-based fibrosis risk indices were.

- Non-alcoholic fatty liver disease fibrosis score (NFS): NFS = –1.675 + (0.037*age[years]) + (0.094* BMI[kg/m^2^] + (1.13*diabetes[yes = 1,no = 0]) + (0.99*AST/ALTratio)–(0.013*platelet[Gpt/l]) –(0.66*albumin[g/dl]). As sensitive/specific cut-off we used, − 1.455 (age 36–65) , 0.12 (age ≥ 65)/0.676 (age ≥ 36), respectively^30^.
- FIB-4-score: FIB-4 = (age[years]_*_AST[U/L])/(platelet[Gpt/l]x(ALT[U/L])^1/2^)^31^. As sensitive/specific cut-off we used, 1.3 (age < 65), 2.0 (age ≥ 65)/2.67 (all ages), respectively^30^.

### Application of guideline recommendations and screening strategies

**Figure 1:**
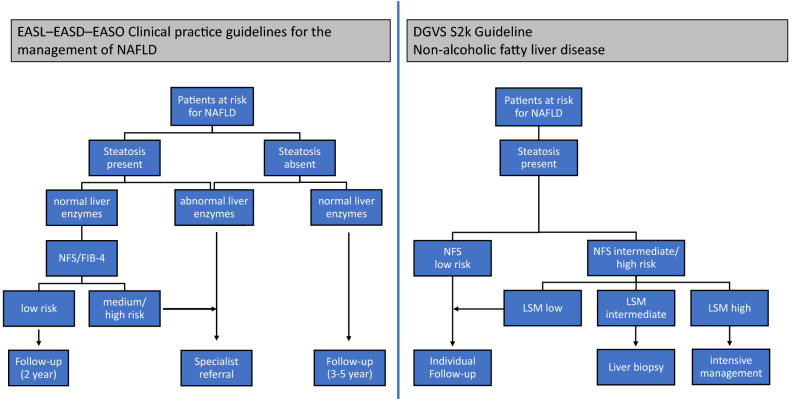
Diagnostic algorithms of guideline recommendations for screening and risk stratification in NAFLD patients^4,18^**.** *NFS* NAFLD Fibrosis score, *FIB-4* Fibrosis-4, *LSM* liver stiffness measurement.

Current international and national guidelines^4,18^ aim to identify patients at risk for fibrosis and suggest different follow-up scenarios or in-depth assessment depending on risk classification, Fig. 1. Because the European Association for Liver/-Diabetes/-Obesity Guidelines (EASL-EASD-EASO) are unspecific regarding the recommended fibrosis scores^4^, we considered all potential variants. National German guidelines do not yet consider age-adapted cut-offs, which we however took into account to improve performance. Patients with increased alcohol consumption were not considered for the main analyses.

Furthermore, we aimed to identify parameters for an optimized referral strategy with reduced referral rates for further hepatological assessment and/or avoiding cost-intensive serum-markers/liver elastography. Based on the results of univariate and multivariate analysis, a simple proposal using AST as a single marker for risk stratification was further evaluated.

### PNPLA3 and TM6SF2 genotyping

In addition to the guideline-based recommendations for NAFLD risk assessment, a genetic analysis of genotyping of common fibrosis risk alleles was performed^32^. In particular, the patatin-like phospholipase domain-containing protein 3 (*PNPLA3*) gene variant p.I148M (*rs738409*) and the transmembrane 6 superfamily 2 (*TM6SF2*) gene variant p. E167K (*rs58542926*) were analysed, see Supplement for details.

### FAST score

Newsome et al. recently evaluated a novel elastography-based scoring system (FAST score) using VCTE to identify patients with active NASH (NASH ≥ 4) and significant fibrosis (F ≥ 2)^22^. The score is calculated from LSM, CAP and AST. At a cut-off of 0.35, the score achieved a sensitivity of ≥ 0.90 for the diagnosis of NASH, a cut-off of 0.67 had a specificity ≥ 0.90^22^. For the present study, FAST score values were retrospectively calculated by the manufacturer of the VCTE device (Echosens, Paris, France).

### Statistical analysis

Statistical analyses were performed using the R statistical package (Version 3.4.2). Means and standard deviations are denoted by X ± Y and medians and interquartile range by X [Y, Z]. LSM was always treated on a logarithmic scale. Group comparisons of continuous variables were based on ANOVA and a chi-squared test without continuity correction was used to assess the association in contingency tables or Fisher’s exact test if expected counts were below 5. Pearson’s linear correlation was used with confidence intervals based on Fisher’s transformation. Linear models were employed to analyse the association between LSM and covariates found to have significant correlations and logistic regression for LSM categories using the established cut-offs^18^, see above. Logistic regression and multivariate analysis were used to identify independent clinical, laboratory data and patients’ characteristics associated with an increased LSM. A p-value < 0.05 was considered significant.

For the EASL-EASD-EASO guideline, LSM categories were used to define the fibrosis risk for calculating diagnostic properties. This cannot be applied to the German guidelines (DGVS) however, which already incorporate LSM. Patients with invalid VCTE examinations were treated as intermediate fibrosis risk with the need of further clarification according to the DGVS definition.

## Results

### Study population

**Figure 2:**
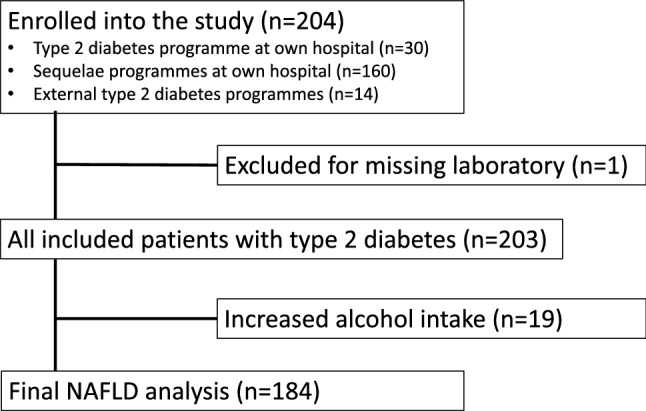
Consort diagram. *NAFLD* Non-alcoholic fatty liver disease.

184 of 204 enrolled subjects qualified for the final analysis (Fig. 2). The baseline characteristics of the study cohort, stratified for LSM, are summarized in Table 1.

**Table 1:** Baseline characteristics, stratified by liver stiffness measurement (LSM).

	Total cohort at NAFLD-risk (n = 184)	LSM low (n = 125)	LSM intermediate (n = 10)	LSM high (n = 46)	p-value	Patients excluded due to alcohol consumption (n = 19)
Number of females	107 (58%)	67 (54%)	6 (60%)	32 (70%)	0.17	2 (11%)
Age (years)	64.2 ± 10.7	64.6 ± 10.7	54.2 ± 7.0	65.5 ± 10.4	**0.0076**	62.6 ± 6.7
BMI (kg/m^2^)	32.6 ± 7.6	31.7 ± 7.0	41.5 ± 11.3	32.3 ± 6.1	** < 0.001**	32.6 ± 6.6
< 25	23 (12%)	15 (12%)	1 (10%)	7 (15%)		3 (16%)
25–30	51 (28%)	42 (34%)	0 (0%)	9 (20%)		5 (26%)
30–35	53 (29%)	37 (30%)	1 (10%)	15 (33%)		4 (21%)
35–40	31 (17%)	17 (14%)	3 (30%)	10 (22%)		5 (26%)
> 40	26 (14%)	14 (11%)	5 (50%)	5 (11%)		2 (11%)
Duration of diabetes (years)	13.0 ± 10.3	12.7 ± 9.9	9.1 ± 5.3	14.5 ± 12.0	0.28	9.8 ± 10.9
Patient reports hepatic problem	86 (47%)	50 (40%)	3 (30%)	32 (70%)	**0.0015**	10 (53%)
HbA1c (%)	6.7 [6.0, 7.6]	6.7 [6.0, 7.6]	7.4 [6.5, 8.1]	6.8 [6.1, 7.8]	0.50	6.3 [5.9, 6.9]
Glucose (mmol/L)	7.3 [6.0, 9.3]	7.0 [6.0, 9.0]	7.7 [6.6, 9.3]	7.8 [6.2, 10.8]	0.17	7.5 [6.3, 8.8]
HOMA-IR	3.9 [1.8, 7.2]	3.3 [1.8, 5.8]	5.4 [2.6, 9.5]	6.3 [2.3, 11.2]	**0.0057**	4.7 [3.2, 7.2]
Triglycerides (mmol/L)	1.8 [1.3, 2.7]	1.8 [1.3, 2.8]	2.1 [1.5, 4.8]	1.7 [1.4, 2.2]	**0.048**	1.6 [1.3, 2.5]
> 1.7	99 (54%)	66 (53%)	7 (70%)	23 (50%)		9 (47%)
HDL (mmol/L)	1.21 [0.96, 1.56]	1.20 [0.97, 1.56]	1.29 [0.99, 1.56]	1.20 [0.93, 1.47]	0.68	1.24 [1.02, 1.48]
low (≤ 1.03)	57 (31%)	36 (29%)	3 (30%)	15 (33%)		6 (32%)
ALT (in units of ULN)	0.76 [0.53, 1.10]	0.69 [0.43, 1.07]	0.91 [0.76, 1.09]	0.94 [0.72, 1.14]	**0.0027**	0.69 [0.55, 0.98]
> ULN	59 (32%)	35 (28%)	4 (40%)	17 (37%)		4 (21%)
AST (in units of ULN)	0.74 [0.52, 0.92]	0.63 [0.48, 0.82]	0.78 [0.61, 0.81]	0.94 [0.75, 1.33]	** < 0.001**	0.71 [0.65, 0.95]
> ULN	38 (21%)	15 (12%)	1 (10%)	21 (46%)		5 (26%)
GGT (in units of ULN)	0.83 [0.50, 1.79]	0.69 [0.39, 1.23]	0.75 [0.68, 1.95]	1.75 [1.01, 3.37]	** < 0.001**	1.14 [0.73, 2.76]
> ULN	78 (42%)	38 (30%)	4 (40%)	35 (76%)		12 (63%)
XL probe used	82 (45%)	50 (40%)	9 (90%)	20 (43%)	**0.0090**	10 (53%)
CAP (dB/m)	309 ± 57	305 ± 54	358 ± 42	309 ± 64	**0.017**	317 ± 57
< 248	21 (11%)	15 (12%)	0 (0%)	6 (13%)		3 (16%)
248–267	16 (9%)	10 (8%)	1 (10%)	5 (11%)		1 (5%)
268–279	14 (8%)	12 (10%)	0 (0%)	2 (4%)		1 (5%)
≥ 280	130 (71%)	88 (70%)	9 (90%)	33 (72%)		14 (74%)
PNPLA3 non-CC (n = 202)	99 (54%)	71 (57%)	8 (80%)	18 (39%)	**0.036**	13 (68%)
TM6SF2 non-CC	155 (84%)	108 (86%)	8 (80%)	36 (78%)	0.41	13 (68%)

Three LSM measurements were invalid. Entries are mean ± standard deviation, median [interquartile range] or numbers (%).

*LSM low* LSM values < 7.9/7.2 kPa M/Xl-probe, *LSM intermediate* 7.9–9.6/7.2–9.3 kPa M/XL-probe, *LSM high* LSM values > 9.6/9.3 kPa M/Xl-probe, *ALT* alanine transaminase, *AST* aspartate aminotransferase, *BMI* body mass index, *CAP* controlled attenuation parameter, *HbA1c* Glycated hemoglobin, *GGT* gamma-glutamyl transpeptidase, *HDL* high-density lipoprotein, *HOMA-IR* homeostasis model assessment of insulin resistance, *NAFLD* non-alcoholic fatty liver disease, *non-CC* non-CC genotype, *ULN* upper limit of normal.

### Guideline recommendations

**Figure 3:**
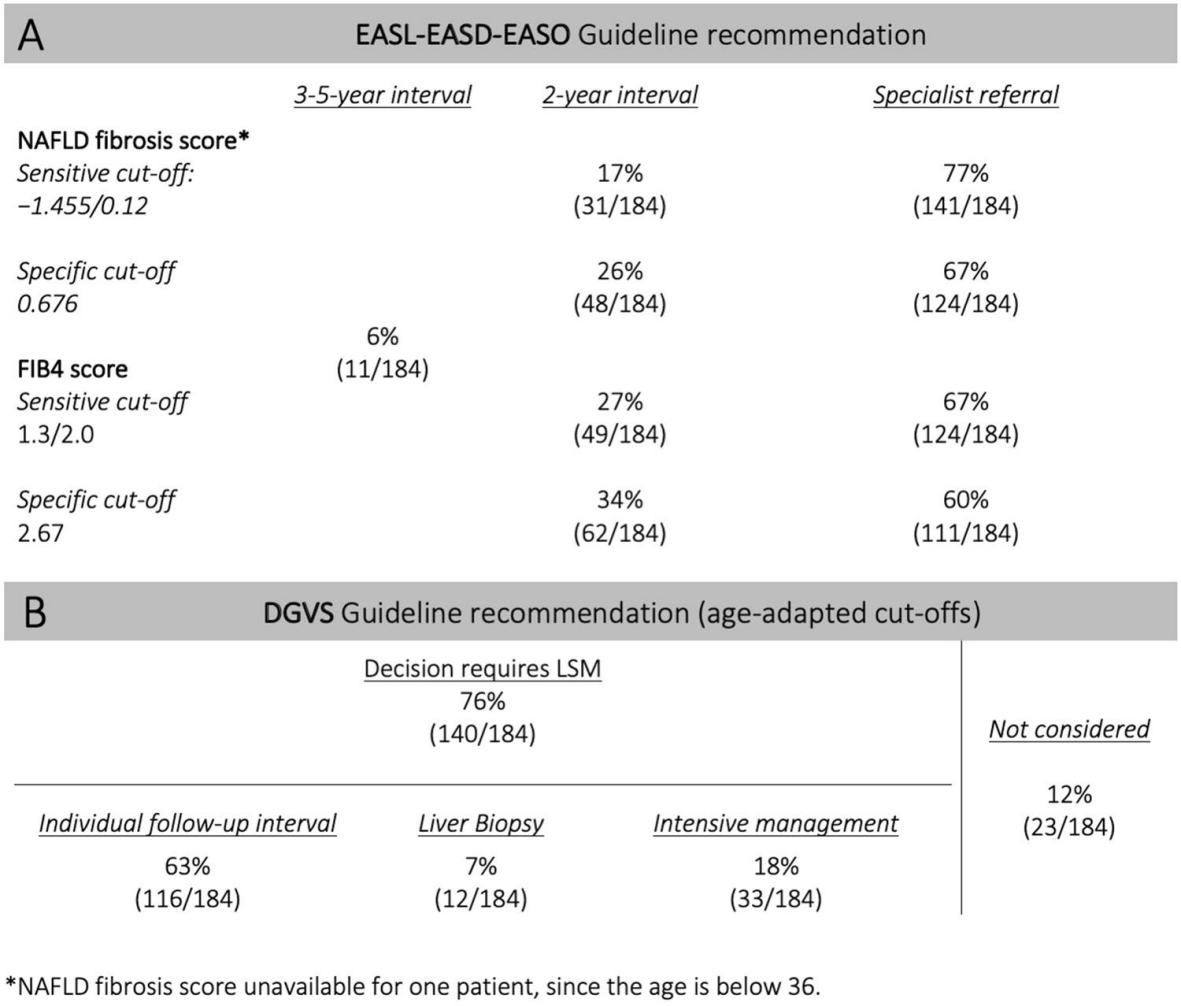
**A**/**B**: Guideline recommendations: clinical consequences and referral rates of diagnostic algorithms proposed by current guidelines in 184 patients with type 2 diabetes and NAFLD. *DGVS* DGVS S2k Guideline non-alcoholic fatty liver disease, *EASL-EASD-EASO* EASL–EASD–EASO Clinical practice guidelines for the management of NAFLD, *LSM* Liver stiffness measurement, *FIB4* Fibrosis-4; age-adapted cut-offs for NAFLD fibrosis score and FIB4 score were used^30^.

Figure 3 shows the results of applying the evaluated guidelines to our study cohort. For the DGVS guideline, 63% of patients are recommended for a long-term follow-up after having been referred to a specialist for LSM. Biopsies are recommended for 7%, and 18% are advised to return to the specialist for intensive management. The remaining 12% do not receive any recommendation although some (n = 9, 5%) have elevated LSM. Three LSM measurements were invalid due to high BMI (38, 43 und 58 kg/m^2^).

**Figure 4:**
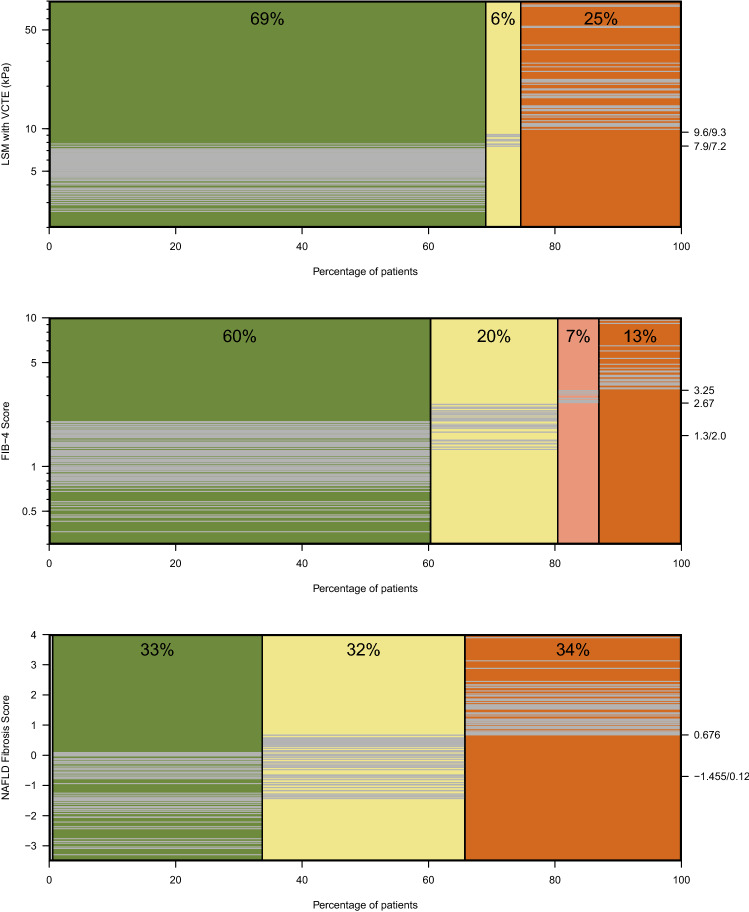
Risk stratification using non-invasive fibrosis scores with established aged-adapted cut-offs (sensitive/specific). The risk categories are colour-coordinated. Each patient’s result is imaged in grey horizontal lines. The more specific cut-off for FIB4 score (3.25) is additionally illustrated; *FIB4* Fibrosis-4, *LSM* liver stiffness measurement, *VCTE* vibration controlled attenuation parameter.

The evaluated guidelines of the EASL-EASD-EASO and the German Society for Digestive and Metabolic Diseases (DGVS) made use of different risk scores (NFS or FIB4) and are unspecific regarding cut-offs (Fig. 4). Hence, we provide the results using different established cut-offs (NFS sens/spec, FIB4 sens/spec^30^). Applying the EASL-EASD-EASO guideline, between 60 and 77% are referred to specialists, 17% to 34% are followed up with 2-year intervals and 6% have long-term follow-up, independent of the risk-score and the cut-offs.

In a second approach we analysed the diagnostic accuracy of applied guideline recommendations according to the NFS and FIB-4 index and variable LSM cut-offs (Table 2). LSM was used as reference for advanced fibrosis for all patients. The German guideline recommendations imply use of the LSM in the diagnostic pathway, thus the PPV was 100% by definition and the number of false positive results cannot be defined. The sensitivity applying the German NAFLD pathway differ from 47–75% in our study cohort. The EASL-EASD-EASO guidelines have a sensitivity ranging from 86 to 96% and a specificity from 30 to 52%, respectively.

**Table 2:** Performance of diagnostic algorithms when applied to patients with type 2 diabetes.

A		LSM	TP	FN	FP	TN	Sens	Spec	Prev	PPV	NPV
EASL-EASO-EASD	*NFS sens*	LSM sens	56	3	85	40	95%	32%	32%	40%	93%
		LSM spec	44	2	97	41	96%	30%	25%	31%	95%
	*NFS spec*	LSM sens	53	6	71	54	90%	43%	32%	43%	90%
		LSM spec	44	2	80	58	96%	42%	25%	35%	97%
	*FIB4 sens*	LSM sens	53	6	71	54	90%	43%	32%	43%	90%
		LSM spec	43	3	81	57	93%	41%	25%	35%	95%
	*FIB4 spec*	LSM sens	51	8	60	65	86%	52%	32%	46%	89%
		LSM spec	41	5	70	68	89%	49%	25%	37%	93%
DGVS	*NFS sens*	defined	44	15	nd	124	75%	nd	32%	nd	89%
	*NFS spec*	defined	28	31	nd	125	47%	nd	32%	nd	80%

B		LSM	TP	FN	FP	TN	Sens	Spec	Prev	PPV	NPV
Simple proposal	*AST* > *ULN for T2D*	LSM spec	21	25	17	121	46%	88%	25%	55%	83%

*LSM* liver stiffness measurement, *TP* true positive, *FN* false negative, *FP* false positive, *TN* true negative, *Sens* sensitivity, *Spec* specificity, *PPV* positive predictive value, *NPV* negative predictive value, *nd* not defined, *T2D* type 2 diabetes, *NFS sens* sensitive cut-off of − 1.455/0.12 (age-adapted) for the NAFLD fibrosis score, *NFS spec* specific cut-off 0.676, *FIB4 sens* sensitive cut-off of 1.3/2.0 (age-adapted), *FIB4 spec* specific cut-off of 2.67, *LSM sens* sensitive cut-off of ≥ 7.9/7.2 kPa (M/XL probe) for Liver Stiffness Measurement, *LSM spec* specific cut-off of > 9.6/9.3 kPa (M/XL probe).

### Alternative proposal for risk stratification

A univariate analysis showed a correlation of NFS with log(FIB4) of 0.79 (95% CI 0.73–0.84). The correlation of NFS with log(LSM) was 0.46 (95% CI 0.33–0.56) and the correlation between log(FIB4) and log(LSM) was 0.52 (95% CI 0.41–0.62).

In a multivariate analysis, LSM was significantly associated with AST and “Patient reports previous hepatic problem”, but not with sex, age, BMI or HbA1c, Table 3 (see supplement 1 for the analogous analysis using LSM categories).

**Table 3:** Multivariate analysis without cut-offs for LSM.

	Estimate (95% CI)	p-value
Sex (male vs female)	0.990 (0.820 to 1.195)	0.91
Age (per year)	1.008 (0.999 to 1.017)	0.096
BMI (per kg/m^2^)	1.004 (0.990 to 1.018)	0.58
HbA1c (per percentage point)	1.015 (0.952 to 1.083)	0.64
Log(AST) (per logULN)	5.363 (3.034 to 9.479)	** < 0.001**
Patient reports previous hepatic problem	1.284 (1.057 to 1.560)	**0.012**

Estimates are n-fold changes in LSM.

Multivariate analysis demonstrates that AST is highly relevant when identifying patients with elevated LSM. Based on this finding, the results using a simple proposal based entirely on AST in type 2 diabetes patients would classify 75% (138/184) for long-term follow-up and the remaining 25% for specialist referral.

Our simple proposal using only the AST > ULN had a sensitivity of 46% with a specificity of 88%. The further diagnostics properties of this proposal are shown in Table 2, Panel B).

### FAST score

**Figure 5:**
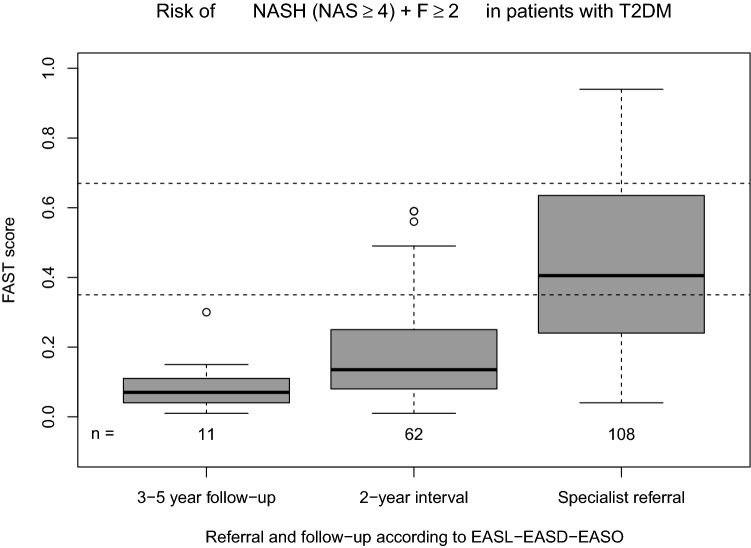
Box plots of FAST score according to EASL-EASD-EASO recommendations. The dashed lines refer to the cut-offs suggested for the FAST score^22^. For risk stratification the specific cut-off of 2.67 were used for FIB4 score^30^.

The application of the recently introduced FAST score using the cut-offs published by Newsome et al.^22^ reduced the required referral to specialist to 35% with the lower cut-off or 12% with the upper one. A sensitivity/specificity calculation was not conducted due to missing reference standard. Figure 5 shows the distribution of the FAST score depending on the recommendation from the EASL-EASO-EASD guidelines. Considering only the patients recommended for specialist referral, we find 45 (42%) below the lower FAST cut-off, 42 (39%) between the two cut-offs, and 21 (19%) above the upper one. The patients above the lower cut-off had slightly higher mean HbA1c (7.3% vs 6.9%, p = 0.18), BMI (33.9 vs 32.5 kg/m^2^, p = 0.30) and duration of disease (13.8 vs 11.1 years, p = 0.19) and age (64.5 vs 61.8, p = 0.17). This provides an indication that these patients may be at higher risk according to known factors as well.

### Genetic analysis for NAFLD risk genes

In addition to guideline recommendations we look at screening markers that may complement screening algorithms in the future. The common NAFLD genetic risk variants of the genes PNPLA3 and TM6SF2 are predestined for such an analysis. The risk variant (non-CC) of the gene PNPLA3 was found in 84 patients and the non-risk variant in 99. There were technical problems with the measurement in 1 case. The risk variant (non-CC) of the gene TM6SF2 was found in 29 patients and the non-risk variant in 155. Both risk variants were not associated with hepatic steatosis according to CAP nor with fibrosis according to NFS. There was a significant association between PNPLA3 and LSM, which did not persist in a multivariate model containing AST. Specialist referral according to EASL-EASD-EASO and DGVS was also not associated with genetic risk variants (see Supplement).

## Discussion

Recommendations for risk assessment in NAFLD patients have not been sufficiently assessed prospectively, in particular in type 2 diabetes. Our population of type 2 diabetics shows (a) high prevalence of elevated LSM indicative of advanced fibrosis, (b) very high referral rates for an in-depth hepatological work-up of up to 77% based on current international and national guidelines and (c) that a much simpler referral algorithm may produce comparably good results.

About 10% of the European general population has diabetes, many of whom have substantial risk for disease progression to NASH and ultimately advanced fibrosis^23,24,33,34^. Liver involvement as a co-morbidity in diabetes has become identified as an important driver of disease progression to NASH or advanced fibrosis with associated complications as hepatocellular carcinoma and is a growing burden to health care. Increased awareness has led to screening guidelines that are unfortunately not sufficiently well-known nor is infrastructure sufficient for their widespread application, especially in primary care^19,21,35,36^. This may prolong the diagnostic process and limit the chances of effective therapeutic interventions before manifestation of cirrhosis or liver cancer^37^.

The optimal screening strategy has excellent sensitivity and specificity, is cheap, widely available, easily administered and gives quick results. In our cohort one quarter of the NAFLD-patients show elevated LSM and thus is at risk for complications of liver fibrosis, a number comparable to what others have found in even larger populations^16,34,38,39^. Optimal screening strategies should identify roughly this number, but the EASL-EASD-EASO guideline leads in our cohort to a referral rate up to 77% and the German guideline requires LSM in 76% of cases. This demonstrates that the diagnostic properties of the algorithms are sub-optimal. Moreover, clarification using LSM is neither cheap due to personnel, nor is it available in primary care. Finally, the step-wise algorithms are not easily administered or understood (see Fig. 1) and, in fact, EASL-EASD-EASO fails to specify the cut-offs for the serum-based fibrosis indices (see Fig. 4).

We identified three major weaknesses in the EASL-EASD-EASO and German algorithms when applied to patients with type 2 diabetes. Firstly, risk stratification according to presence of steatosis using conventional ultrasound identifies almost the entire population, but overlooks patients requiring attention in the small number excluded. These may be patients with advanced disease whose steatosis has already begun to recede (9/23 in our data). Second, the application of the original NFS without age-adaptation categorizes almost all diabetics to be “at risk” and seems unsuitable for application in the obese diabetic cohort. Even with the age-adapted cut-offs used here, NFS remains quite unspecific. On the other hand, the FIB-4 score seems promising with the age-adaptation. Unfortunately, there are many suggestions for cut-offs with little consensus (cf. Fig. 4 and e.g. the cut-offs at 2.67 and 3.25). Finally, NAFLD requires the exclusion of all other potentially relevant diseases including even fairly moderate alcohol consumption^4,18^. In our cohort, 19 patients were excluded based on this definition, 12 of whom consumed only up to two drinks per day, and half of whom are at risk for advanced fibrosis. Such alcohol consumption may well aggravate the diabetic effect on liver disease but falls between the cracks of disease definitions and guidelines. Self-reporting on alcohol consumption is known to be unreliable^40^ and it could be more sensible to include patients with moderate alcohol consumption for secondary preventive measures.

Liver stiffness can be considered the gold standard of non-invasive risk assessment, with VCTE the best evaluated and approved method for NAFLD^13,14^. A particularity of the German guideline is the implementation of VCTE in the screening algorithm, which was indicated in about 75% of our patients. LSM-based approaches were recently evaluated from a health economics standpoint and found to be potentially cost-saving in populations at risk^41^. However, universal access to this method would require large investments^21^ and modifications to the reimbursement system in most European countries, depending on national health care. Although this works in tertiary referral centres, extrapolation indicates that the method’s PPV could be a serious limitation in primary care^16,42^.

Our prospective data corroborate retrospective ones, suggesting that the EASL-EASO-EASL algorithms are not easily implementable in the outpatient healthcare^12^. Moreover, they agree with a study showing that the high prevalence of liver steatosis constitutes a problem for the algorithms^43^. Hence, we see the need for very low threshold identification strategies based on simple reliable patient characteristics and low-cost laboratory data, e.g. BMI, AST and sex. Our multivariate analysis demonstrates that diabetic patients with elevated AST are prone to increased LSM. A simple proposal based exclusively on elevated AST in type 2 diabetics would be easy to implement and have acceptable diagnostic properties despite sending “only” 25% to liver specialists. Recent publications have also found AST to be a simple marker for fibrosis^44^ and that there is an association with progression of fatty liver disease^45^.

Genetic testing for risk alleles were in line with known distribution of disease severity^32,46–48^, but relative risk was too low to suggest a meaningful diagnostic benefit.

The guideline recommendations were developed to identify patient at risk for liver fibrosis because of its association with liver specific morbidity and overall mortality^5,7,49^. Ongoing phase 2 and 3 trials in drug development for the therapy of NASH suggest that medication will soon be available to prevent fibrosis progression in NASH^50^. Future screening strategies will include NASH, a condition which could not be identified without liver biopsy until now. The recently proposed FAST score is a composite of VCTE and AST and could be helpful for stratification of treatment decisions^22^. The application to our cohort had a favourable distribution and especially patients with a high score and recommended for specialist referral by the EASL-EASO-EASL algorithm may be interesting candidates for pharmacological therapy, but requires further evaluation. Beyond the guideline based recommendations, many serum markers such as M2BP, hyaluronic acid or type IV collagen 7 are being evaluated for fibrosis detection in different settings^51^.

We recognize that the missing gold-standard by histological proven diagnosis is a limitation. However, biopsies are often contraindicated in diabetes patients with cardiovascular comorbidities requiring anti-coagulation and/or antithrombotic therapy. Hence, to avoid selection bias, suitable surrogates such as VCTE represent the best available option^16^.

Even assuming a PPV as low as 50%, our data suggest advanced fibrosis in 12% of the cohort. While our cohort may have more severe disease than the general diabetic population, this bias is shared by almost all studies as it is inherent to a university hospital. Hence, our single centre study should be reproduced in a multi-centre approach, ideally in primary care. Such a study should be accompanied by a detailed health-economics analysis to verify potential cost benefits of such screening strategies^41^.

With increasing NAFLD prevalence, efficient screening strategies are of the utmost importance. The DGVS and EASL-EASD-EASO guidelines constitute an important starting point, but show high referral rates to liver specialists in diabetic populations and require prohibitively substantial resources. Greater focus on AST and less focus on conventional ultrasound and complex algorithms may be the next steps toward improvement.

## Supplementary information

Supplementary Information

## Data Availability

The study data are available on request. Please contact Thomas Karlas (thomas.karlas@medizin.uni-leipzig.de).

## Abbreviations

ALT: Alanine aminotransferases
AST: Aspartate aminotransferases
BMI: Body mass index
CAP: Controlled attenuation parameter
DGVS: German Society for Digestive and Metabolic Diseases
EASL: European Association for the Study of the Liver
EASO: European Association for the Study of Obesity
FAST: FibroScan-AST
FN: False negative
FP: False positive
GGT: Gamma-glutamyl-transferase
HbA1c: Glycohemoglobin
HOMO-IR: Homeostasis model assessment of insulin resistance
LSM: Liver stiffness measurements
NAFLD: Non-alcoholic fatty liver disease
NASH: Non-alcoholic steatohepatitis
Nd: Not defined
NFS: Non-alcoholic fatty liver disease fibrosis score
NPV: Negative predictive value
PPV: Positive predictive value
PNPLA3: Patatin-like phospholipase domain-containing protein 3
Sens: Sensitivity
Spec: Specificity
TM6SF2: Transmembrane 6 superfamily 2
TN: True negative
TP: True positive
ULN: Upper limit of normal
VCTE: Vibration controlled transient elastography
