# Supplementary material for: Current NAFLD guidelines for risk stratification in diabetic patients have poor diagnostic discrimination
Source: Sci Rep. 2020 Oct 27;10:18345. doi: 10.1038/s41598-020-75227-x (PMC7591877; doi:10.1038/s41598-020-75227-x)
Supplement: Supplementary file 1 — Supplementary Information [file 41598_2020_75227_MOESM1_ESM.docx]

**Current NAFLD guidelines for risk stratification in diabetic patients have poor diagnostic discrimination.**

Blank Valentin**^1,2^, Petroff David**^2,3^, Beer Sebastian^1^, Böhlig Albrecht^4^, Heni Maria^1^, Berg Thomas^4^, Bausback Yvonne^5^, Dietrich Arne^2,6^, Tönjes Anke^7^, Hollenbach Marcus^1^, Blüher Matthias^2,7^, Keim Volker^1^, Wiegand Johannes^4^, Karlas Thomas*^1^

1. Division of Gastroenterology, Department of Medicine II, Leipzig University Medical Center, Liebigstraße 20, 04103 Leipzig, Germany
2. Integrated Research and Treatment Center (IFB) AdiposityDiseases, University of Leipzig, Philipp-Rosenthal-Str. 27, 04103 Leipzig, Germany
3. Clinical Trial Centre Leipzig, University of Leipzig, Härtelstraße 16/18, 04107 Leipzig, Germany
4. Division of Hepatology, Department of Medicine II, Leipzig University Medical Center, Liebigstraße 20, 04103 Leipzig, Germany
5. Division of Angiology, Leipzig University Medical Center, Liebigstraße 20, 04103 Leipzig, Germany
6. Division of Visceral, Transplantation, Thorax and Vascular Surgery, Section of Bariatric Surgery, Leipzig University Medical Center, Liebigstraße 20, 04103 Leipzig, Germany
7. Division of Endocrinology and Nephrology, Leipzig University Medical Center, Liebigstraße 20, 04103 Leipzig, Germany

**the authors contributed equally to the work *corresponding author

*Genetic methods*

Genomic DNA was extracted from peripheral blood leukocytes according to standard manufactural protocol (QIAamp DNA Blood Mini Kit, Qiagen, Germany). Polymerase chain reaction was performed using OneTaq 2 x Master Mix with standard buffer, 100 nM of each primer and 1 µl genomic DNA in a total volume of 25µl. Cycle conditions were an initial denaturation for 10 minutes at 95°C followed by 40 cycles of 30 seconds denaturation at 95°C, 30 seconds annealing at 60°C, 60 seconds primer extension at 72°C and a final extension for 10 minutes at 72°C in an automated thermal cycler (Thermocycler PTC-200, MJ Research, Watertown, MA, USA). Primers were synthesized according to the published nucleotide sequences: *PNPLA3* (NCBI: NC_000022.11) Forward-Primer 5’-CTTSTGAAGGATCAGGAAAATTAAA-3’, Revers-Primer 5’-GGGACAGACCTGAGGT-3’; *TM6SF2* (NCBI: NC_000019.10) Forward-Primer 5’-AGCATGGCACCAGCAGGTA-3’, Reverse-Primer 5’-CAAACAGATGTCCAGCAGGGT-3’.

We performed melting curve analysis in the LightCycler 480 instrument (Roche Diagnostics, Mannheim, Germany) using fluorescent resonance energy transfer (FRET) probes. FRET probes were designed complementary to the mutated sequence. Probes were devised and synthesised by TIB Molbiol (Berlin, Germany). *PNPLA3* variants were determined by using an anchor probe 5’-ACCACGCCTCTGAAGGAAGGAGGGATAAG-FL-3’ and sensor probe 5’-LC610-CCACTGTAGAACGGCATGAAGC--PH for hybridization, following denaturation at 95°C for 60 seconds, starting from 40°C to 80°C. *TM6SF2* variants were analysed by hydrolysis starting from 45°C to 70°C using a single probe 5’-AAGGCAAXITACAGCTCCAAGATCAGG--PH.

**Supplement 1: Logistic regression analysis based on cut-off values for LSM recommended by the German society for gastroenterology (DGVS).**

| LSM cut-off | 7.9 kPa / 7.2 kPa (sensitive) | | 9.6 kPa / 9.3 kPa (specific) | |
| --- | --- | --- | --- | --- |
|  | Estimate (95% CI) | p-value | Estimate (95% CI) | p-value |
| Sex (male vs female) | 0.637 (0.296 to 1.339) | 0.24 | 0.699 (0.305 to 1.568) | 0.39 |
| Age (per year) | 1.000 (0.965 to 1.038) | 0.98 | 1.019 (0.980 to 1.062) | 0.35 |
| BMI (per kg/m^2^) | 1.046 (0.996 to 1.100) | 0.76 | 0.983 (0.924 to 1.041) | 0.57 |
| HbA1c (per percentage point) | 0.885 (0.680 to 1.130) | 0.34 | 0.927 (0.683 to 1.216) | 0.60 |
| logAST (per logULN) | 12.3 (4.41 to 38.9) | **<0.001** | 9.37 (3.39 to 29.5) | **<0.001** |
| Known chronic LD  (vs without) | 1.539 (0.734 to 3.240) | 0.25 | 2.919 (1.304 to 6.796) | **0.010** |

*Genetic risk variants*

PNPLA3: The risk variant (non-CC) of the gene PNPLA3 was found in 84 patient and the non-risk variant in 99. There were technical problems with the measurement in 1 case. This distribution is compatible with the Hardy-Weinberg equilibrium, p=0.33. The mean CAP value was 311 dB/m for the risk variant vs 308 for the non-risk variant (95% CI for the difference –14 to 19 dB/m, p=0.75). The mean NFS was 0.25 in the risk vs –0.05 in the non-risk variant (95% CI for the difference –0.10 to 0.69, p=0.14). For LSM, the geometric mean value is 8.1 kPa for the risk group vs 6.4 for the non-risk group (95% CI for the factor difference 1.04 to 1.56, p=0.018). If one is a carrier of the risk allele, then the probability that LSM is elevated according to guideline definitions [18] (7.9 kPa for the M-probe and 7.2 kPa for the XL probe) is 35% compared to 27% otherwise, p=0.24. However, carriers of the risk allele have a probability of 33% compared to 19% for if having highly elevated LSM (9.6 kPa for the M-probe and 9.3 kPa for the XL probe), p=0.031. This effect persisted in a multivariate logistic regression containing age, sex and BMI, but not if AST (elevated/not elevated) was included. TM6SF2: The risk variant (non-CC) of the gene TM6SF2 was found in 29 patient and the non-risk variant in 155. This distribution is compatible with the Hardy-Weinberg equilibrium, p=0.37. The mean CAP value was 303 dB/m for the risk variant vs 310 for the non-risk variant (95% CI for the difference –27 to 12 dB/m, p=0.45). The mean NFS was 0.05 in the risk vs 0.35 in the non-risk variant (95% CI for the difference –0.29 to 0.90, p=0.31). For LSM, the geometric mean value is 8.1 kPa for the risk group vs 7.0 for the non-risk group (95% CI for the factor difference 0.84 to 1.60, p=0.35).
